# Conducting spinal cord injury model with clip compression in rodents: Pearls and pitfalls

**DOI:** 10.1016/j.mex.2023.102231

**Published:** 2023-05-26

**Authors:** Arman Vahabi, Anıl Murat Öztürk

**Affiliations:** Department of Orthopaedics and Traumatology, Ege University School of Medicine, Izmir, Turkey

**Keywords:** Clip Compression Model for Spinal Cord Injury, Rat, Rodent, Spine injury, Nerve injury, Animal experiment

## Abstract

Research on spinal cord injuries is an important and living topic that raises many critical questions that need to be addressed. While numerous articles have compiled and compared various models of spinal cord injuries, there is limited comprehensive guide with clear instructions available for researchers who are unfamiliar with clip compression model. This model creates acute compression damage in spinal cord, which aims to mimic the nature of traumatic spinal cord damage in humans. Purpose of this article is to share our experience on clip compression model, with experience gained from more than 150 animals, and to provide guidance for researchers with lack of experience who wish to design studies with this model. We have defined several key variables, as well as the difficulties that may arise when applying this model.

− Proper preparation, good infrastructure and necessary tools and knowledge of anatomy related is essential to the success of this model.
− Good exposure with non-bleeding surgical site is key factor for surgical step.
− Postoperative care is particularly challenging, and researchers should consider extending their studies over a reasonable time period to ensure that appropriate care could be provided.

## Graphical abstract

**Figure:**
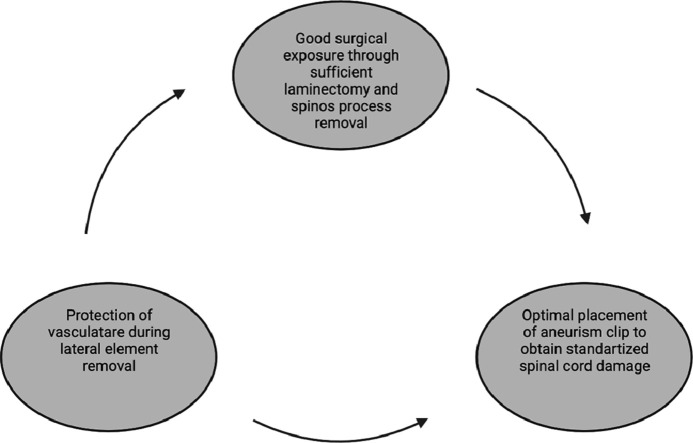

Specifications table

**Table:** 

Subject area:	
More specific subject area:	Spinal Cord Injury Models
Name of your method:	Clip Compression Model for Spinal Cord
Name and reference of original method:	[1]
Resource availability:	https://www.aesculapusa.com/en/healthcare-professionals/or-solutions/aneurysm-clips-for-neurosurgery.html http://www.xenosys.co.kr/main/main.html#sec_1 https://www.georgetiemann.com/layout.asp https://www.integralife.com/heiss-automatic-skin-retractor/product/surgical-instruments-hospitals-surgery-centers-tissue-banks-integra-ent-otology-retractors-heiss-automatic-skin-retractor

## Method details

### Preoperative considerations

**Fig. 1:**
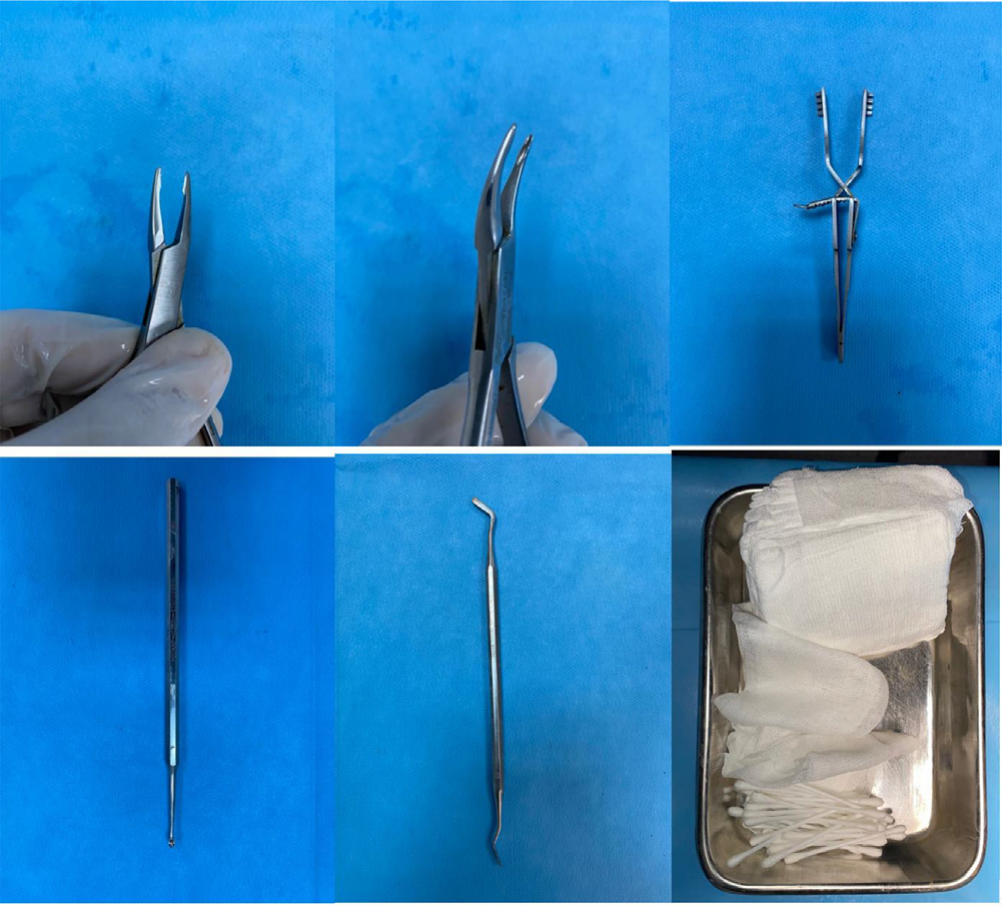
Necessary tools for this model. 1A: straight-ended micro-rongeur 1B: curve-ended micro-rongeur 1C: automatic skin retractor 1D: micro-curette 1E: Woodman retractor 1F: autoclaved gauzes and cotton-swabs.

The following tools are necessary for conducting this animal model: Scalpel, No. 11 blade, micro-curette, automatic blunt skin retractor (4 inches), micro-Adson forceps, straight-ended micro-rongeur, curved-ended micro-rongeur, and Adson forceps, Woodson elevator (for tissue sampling) micro-scissor, scissor and a aneurism clip of choice. We used autoclaved cotton-swabs and gauzes. Do remember cotton-swabs are very practical solution for intraoperative bleedings. (Fig. 1)

Several authors have reported using a 10x magnifying surgical microscope during their surgical procedures. However, despite having the 10x surgical microscope, we opted to use 5x magnifying surgical loupes for our procedures. We believe that the 5x magnifying surgical loupes provide sufficient magnification with clear visibility of all related structures, while also being more practical to use than the surgical microscope.

Post-shipping acclimatization is one of fundamental steps for animal experiments.In our settings, rats were bred in the same facility, eliminating the need for us to wait for post-shipping acclimatization. But this surgery is a big insult for animal's well-being and details like that becomes more important so researchers must follow this basic rule. We had the infrastructure to provide either ketamine-xylazine anesthesia or inhaled isoflurane anesthesia. We chose to conduct our study with ketamine-xylazine anesthesia due to its ease of use in our settings. Application of ketamine/xylazine anesthesia (40–90 mg/kg ketamine + 5–10 mg/kg xylazine) provides anesthesia duration of 45 to 90 min, which is sufficient for this procedure in experienced hands. For researchers without experience, inhaled anesthesia may be a better choice initially, as duration of anesthesia can be extended more easliy in case of need.

While some authors have described this procedure in mice, there are concerns about the spontaneous healing capacity of the mouse spinal cord injury and the transferability of data obtained from mice to humans. Therefore, the vast majority of studies conducted in this model have used rats, which we also preferred to use in our study. We did not select a specific gender in our study, used both male and female Sprague-Dawley rats aged between 8 and 12 weeks with a weight range of 240–420 gs, depending on their gender [2], [3], [4].

Paracetamol, tramadol, or ketoprofen can be considered as the analgesic of choice for this model. While there may be some concerns about the use of non-steroidal anti-inflammatory agents, as they can interfere with various pathways and potentially affect the study's results in some studies, one must not forget that this procedure is a significant insult to the animal and the basic analgesic agents’ effect may not be sufficient.

### Operative considerations

In animal studies, it is crucial to apply a standardized procedure as much as possible. When it comes to this model, several variables require full attention, as they could significantly affect the results obtained.

These variables include:

• The extent and level of paraspinal muscle detachment from posterior elements.
• The extent and level of laminectomy and removal of spinal processes.
• The placement of the clip in optimal position as it might damage different parts of the cord.

**Fig. 2:**
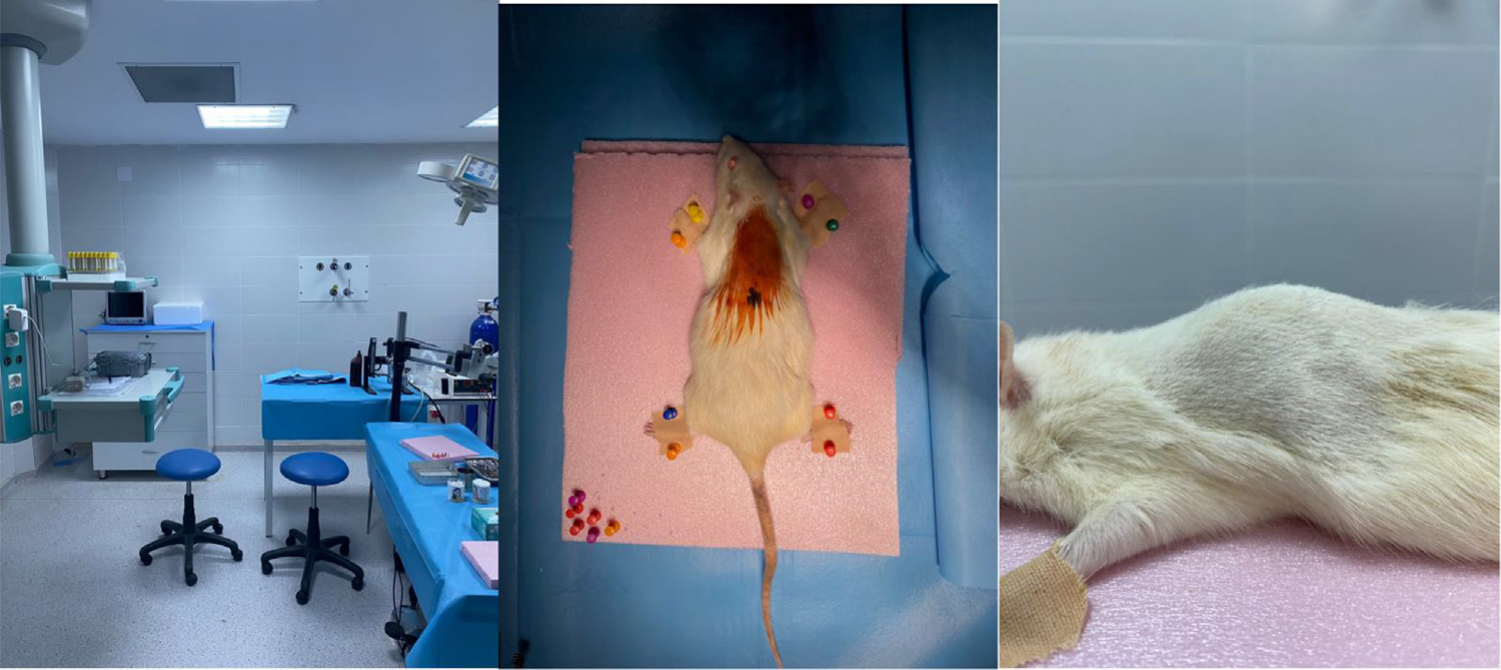
Preoperative preparation before surgical step. 2A: surgical Table 2B: placement and fixation of rat on Table 2C: lateral view of operative area underlining the prominence of T13 which should be used as surgical landmark.

Once the animal is prepared and in place, the level of damage to be inflicted must be identified. Majority of researchers prefer to use the T8-T10 level, as it results in paraplegia and allows for monitoring of healing through improvements in lower limb control. We also chose this level to create the damage, using the prominence of the T13 vertebra as a landmark. The skin incision starts from this prominence and extends cranially by about 3 cm. The midpoint of this line coincides with the T8-10 level. The next step is to detach the paraspinal muscles from the posterior elements. In our opinion, this stage presents several pitfalls, and avoiding them is the crucial first step in achieving good exposure (Fig. 2).

To begin the detachment of the paraspinal muscles from the posterior elements, the spinous processes should first be identified, and this line should be used as a reference. Then using a No. 11 blade, make a sharp first move on both sides of spinous processes. After this initial sharp detachment, the detachment should be completed bluntly with the help of a micro-curette. With practice, this technique can be mastered, and three levels of muscle detachment should be sufficient for achieving good exposure. Once sufficient detachment of the paraspinal muscles has been achieved, a blunt skin retractor can be placed at this layer, allowing for the next step to be carried out.

At this stage, you will be facing the posterior elements of the vertebral column. After double-checking your level, proceed with the laminectomy and removal of the spinous processes. The first step in laminectomy is the hardest since you need to create an appropriate cleavage to obtain sufficient laminectomy. As technical trick, we suggest grabbing and slightly elevating the vertebral column from a remote cranial level so that the distance between the vertebrae increases, allowing for easier access to the epidural space.

Start the laminectomy by removing the spinous process at the level of intended damage with a straight-ended or curved-ended micro-rongeur. After this initial step, look for any cleavage for your curved-end micro-rongeur. Flexion-extension moves, with the help of the grabbed column, might aid in choosing an entry point for your cleavage. Throughout all the steps regarding the laminectomy, it is crucial to prevent continuity of the dura since our model aims to create an extradural compression. Any damage to the dura would render the animal unsuitable for the study.

**Fig. 3:**
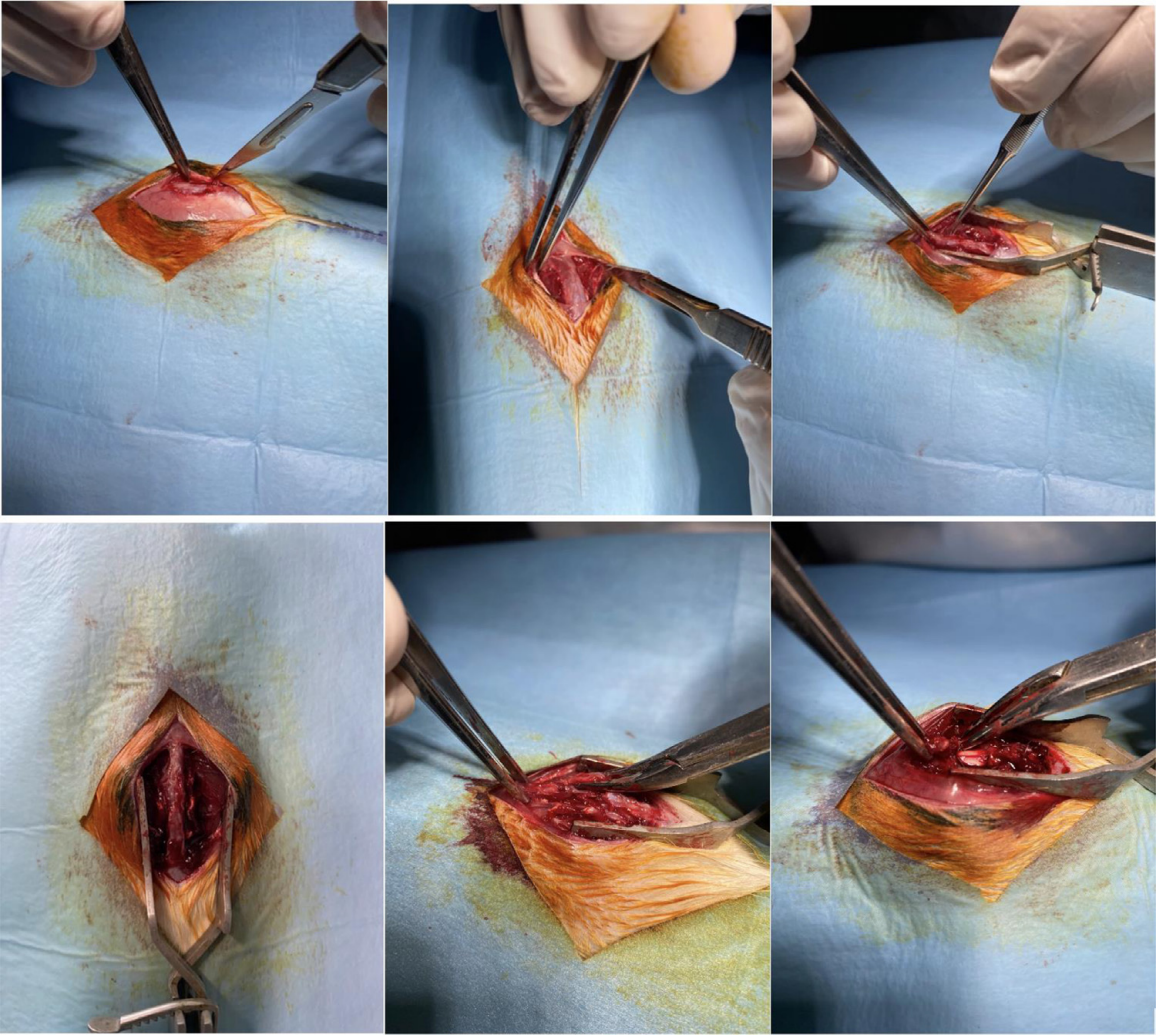
Surgical steps 3A: dissecting subcutaneous layers 3B: sharp detachment of paraspinal muscles 3C: blunt detachment of paraspinal muscles with help of micro-curette 3D: final exposure before removal of posterior elements 3E: first bite of laminectomy, pay attention to distant grabbing of vertebral column 3F: exposure of spinal cord after completion of laminectomy.

**Fig. 4:**
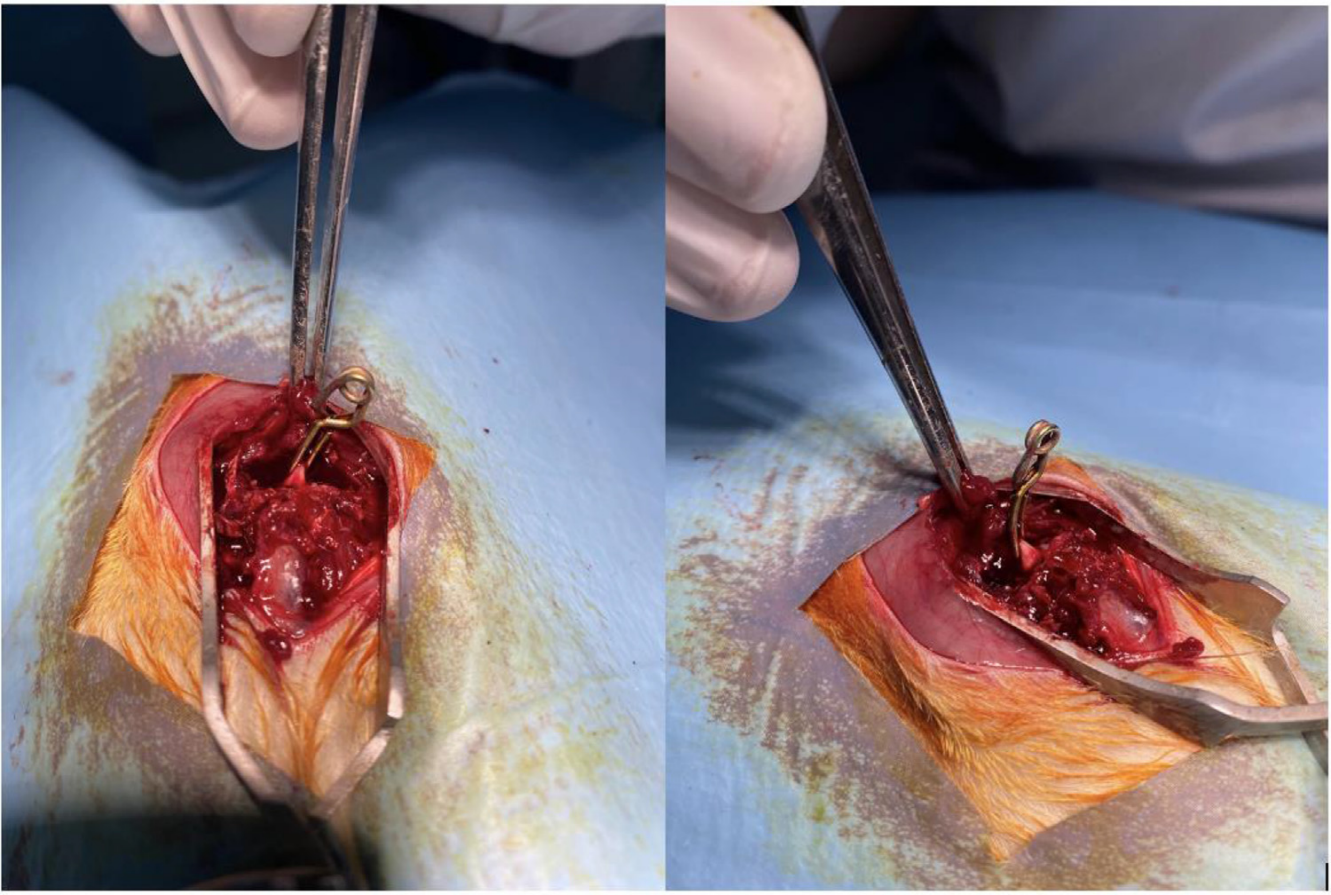
Application of aneurism clip. Note that clip grabs the whole cord that allows it to compress all spinal tracts homogenously.

After the first bite with rongeur, completing the laminectomy at the desired level becomes easier. However, there lies perhaps the biggest pitfall of this model. Performing a laminectomy solely on posterior structures will make the spinal cord clearly visible, but it will not allow for the placement of the clip since there needs to be space on the lateral sides for the clip to fully compress the cord. Failure to remove some lateral elements will result in either insufficient compression of the cord (as only the posterior tracts will be compressed) or no compression at all, as the clip cannot be placed properly (Figs. 3,4).

One might think that it would be a good idea to remove all the lateral elements and place the clip as desired. However, the problem here is that the vascular structures running on both sides of the vertebral column are susceptible to damage during the cleaning of the lateral elements. Bleeding from these vessels not only creates additional surgical stress for the animal but also reduces the quality of exposure of the visibility surgical field, making the later stages of the procedure more difficult. Some of these bleedings can be managed by compression with the help of cotton swabs, while some excessive bleedings may be responsible for intraoperative and early postoperative mortalities. Therefore, it is crucial to handle the lateral structures with great care and avoid any unnecessary damage to the vascular structures.

The placement of the aneurysm clip is next stage of the procedure. The clips come in various lengths, shapes (curved or straight), and applies different pressures. Some researchers have compressed the cord with a clip for 30 seconds while others have applied it for 60 seconds. Some use clips with 30 g pressure while some uses clips with 50 g pressure. These variables can be adjusted based on the specific characteristics of the study. However, it is important to standardize the procedure. On each animal, the clip must catch the cord medio-laterally and compress the entire cord circumferentially and should be held in place for the same duration. Once proper placement is confirmed, a chronometer should be started since it may need to be readjusted in the case of insufficient lateral element exposure (Fig. 4).

It is important to consider that these clips have not been designed for multiple applications and reuse. Particularly in studies with larger sample sizes, the compression force of the clip may decrease with repetitive use. While it is not practical to apply new clips to every animal due to the associated costs, there is currently no practical method to determine if a clip is still applying sufficient pressure. To address this issue, we utilize several methods. Firstly, we used artery that runs on the dorsal side of the cord as an indicator as blood flow in this artery is clearly visible. Compression with clip should stop the blood flow. Failure to observe this change should indicate malfunctioning clip. Other way to evaluate clips function is to observe ecchymosis at the site of compression. Visible ecchymosis should be present after removing the clip, and failure to produce such ecchymosis could indicate a malfunctioning clip. Another variable that can be observed is the clinical outcome of the clip compression. After index surgery, we expect animals to be paraplegic and to have no bladder control. Failure to achieve these outcomes postoperatively could again indicate a malfunctioning clip. Although there are several factors that can affect the effectiveness of the clips, we recommend changing the clip every 50 animals or so.The final and crucial stage is the removal of the clip. If the clip is retracted without being fully opened, it can potentially damage the dura and lead to the exclusion of the animal from the study.

### Postoperative considerations

We have observed that rats that experienced intraoperative bleeding had a higher mortality rate. Therefore, it is crucial to avoid damaging vascular structures during exposure. Although we provided all animals with subcutaneous serum injections in postoperative period, we still experienced early postoperative mortalities initially which we believe some of them were related to hypovolemic shock. Close monitoring is highly recommended to identify animals that require additional hydration.

Postoperative recovery rates can vary greatly between animals. It is important to identify and closely observe animals that do not begin oral intake to prevent avoidable moratalities. Another important rule is to keep each animal in a separate cages postoperatively. Once recovered, animals may try to bite each other's wounds, which could potentially be morbid to animals at this vulnerable stage. While studies with large numbers of animals that require a significant number of cages could tempt researcher to break this rule, it is advisable to avoid this issue by extending their study period. We have also observed that, after becoming paraplegic due to the described technique, animals may even attempt to bite or eat their own extremities. Therefore, it is crucial to monitor animals closely to prevent self-injury. Appling daily povidone-iodine to wound and extremity (in case of need) is feasible solution for this kind of problem.

**Fig. 5:**
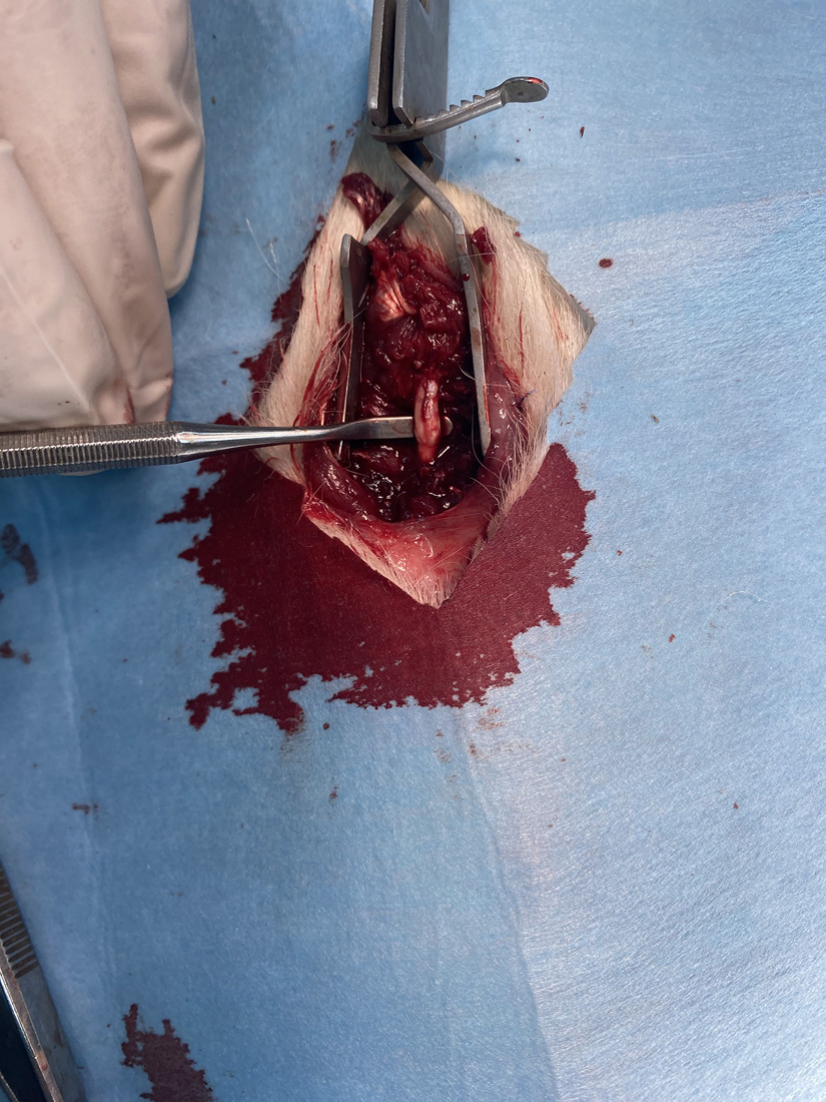
Tip we recommend for tissue sampling. Note extended laminectomy that is also necessary for optimal tissue sampling.

The surgical site created during the index surgery may not be sufficient for tissue sampling, as most studies have reported collecting spinal cord samples measuring 1 to 2 cm in length. After exposing the damaged area of the cord, it is necessary to extend the laminectomy both proximally and distally, while keeping in mind the apex of the compressed level. It is important to be aware of the spinal cords fatty structure, as there are only one or two chances to obtain a clean edge tissue sample. We have developed a trick to avoid this pitfall: after extending the laminectomy, we use a Woodson elevator to go under the spinal cord (anterior), and then make a sharp cut with scissors at both ends, which has proven to be an effective way to collect tissue samples (Fig. 5).

Based on our experience, we have found that obtaining blood samples from the tail vein becomes extremely challenging after inducing spinal cord injury and subsequent paraplegia. While we were unable to confirm this observation in our literature search, we believe that future studies should be aware of this potential issue. To mitigate this problem, it may be advisable to schedule blood sample collection around the time of euthanasia to avoid difficulties associated with tail vein access.

Postoperative care primarily focuses on wound management, bladder massage, and monitoring vital signs. The oral intake of the animal serves as a good indicator of its overall well-being, and vice versa. Adequate amounts of soft bedding should be provided to prevent pressure wounds, particularly during the early postoperative days. Bladder massage should be performed at least twice daily during the first few days, and although some animals may regain bladder control over time, daily monitoring for their bladder function and wound is still necessary. Some animals may exhibit hematuria and pyuria. In such cases, additional antibiotic doses and more frequent bladder massages are administered to address these issues.

## Ethics statements

We confirming that those experiments complied with the ARRIVE guidelines and were carried out in accordance with the U.K. Animals (Scientific Procedures) Act, 1986 and associated guidelines; EU Directive 2010/63/EU for animal experiments; or the National Institutes of Health guide for the care and use of laboratory animals (NIH Publications No. 8023, revised 1978).

Animals are included to study regardless of their gender. It was not predicted that the variables we studied in our projects would be affected by sex hormones.

## Funding

Research projects that provided the authors with the experience discussed in this article were supported by the Ege University Scientific Research Committee (TGA-2021-23305).

## CRediT authorship contribution statement

**Arman Vahabi:** Conceptualization, Methodology, Writing – original draft. **Anıl Murat Öztürk:** Supervision, Writing – review & editing.

## Declaration of Competing Interest

The authors declare that they have no known competing financial interests or personal relationships that could have appeared to influence the work reported in this paper.
